# On the role of mutualisms in plant biogeography: consequences for ecology, evolution, and invasion

**DOI:** 10.1111/nph.70465

**Published:** 2025-08-09

**Authors:** Camille S. Delavaux

**Affiliations:** ^1^ Institute of Integrative Biology ETH Zurich (Swiss Federal Institute of Technology) Universitätsstrasse 16 8092 Zurich Switzerland

**Keywords:** biogeography, invasion, mutualism, mycorrhizal fungi, nitrogen‐fixing bacteria, plants, pollinators, seed dispersers

## Abstract

Most plant species world‐wide depend on one or more mutualisms – beneficial associations with other species. Evidence is emerging that these biotic mutualisms shape plant biogeography (i.e. distributions). In particular, the absence of these mutualist partners limits plant establishment (i.e. the mutualist filter). Moreover, this mutualism filter has subsequent consequences for plant ecology, evolution, and invasion. However, a review of such evidence and a synthesis of mechanisms underpinning the mutualist filter are lacking. Therefore, here, I present evidence for the mutualist filter, discuss ecological and evolutionary consequences of this filter, and develop a synthetic framework, generating several cross‐mutualist predictions of mutualist filter strength. The mutualist filter should increase with higher mutualist dispersal limitation, specificity and dependency of association, and an increasing number of mutualisms. Together, this offers a path to shift our abiotic and antagonistic centric perspective in plant biogeography to integrate these pervasive mutualistic biotic interactions.

**Table:** 

	Contents	
	Summary	714
I.	Introduction	714
II.	Mutualist filters in island floras	715
III.	Implications for ecology and evolution	717
IV.	Consequences for invasion risk	717
V.	Traits influencing the mutualist filter: a synthesis	718
VI.	Conclusions	719
	Acknowledgements	719
	References	719

## I. Introduction

Plant biogeography – the study of plant distributions and underlying drivers – has historically focused on environmental, abiotic drivers. Accordingly, classically studied drivers of plant biogeography include latitude, climate, and geological history (MacArthur & Wilson, 1967; Kreft & Jetz, 2007; Losos *et al*., 2010; Whittaker *et al*., 2017). Over the past decades, a growing body of work has begun to integrate biotic drivers of plant distributions, with a strong focus on antagonistic interactions (Wiens, 2011). This includes the impacts of herbivory and pathogens on the maintenance of plant diversity and generation of biogeographical patterns (Janzen, 1970; Connell, 1971; Wiens, 2011; Bever *et al*., 2015). However, most plant species rely on mutualisms – beneficial associations with other species (Box 1). In fact, emerging work places mutualisms as key drivers in determining macroecological patterns of plant diversity and distributions (Taylor *et al*., 2019; Delavaux *et al*., 2024). Determining the relative importance of these abiotic and biotic drivers will be key to a predictive understanding of plant biogeography.

Box 1 **Definitions**

*Plant biogeography* – The study of patterns and drivers underpinning plant distributions.

*Mutualisms* – Beneficial associations with other species, often with organisms in distinct kingdoms.

*Plant mutualisms* – Mutualisms between plants and other organisms; the major types of plant mutualism include defense, nutritional, and dispersal mutualisms.

*Mutualist filter* – Plant establishment is in part determined by the availability of mutualists; a reduced availability of mutualists serves as a filter to associated plants.

*Specificity (plant)* – Level of plant selectivity in the mutualism.

*Dependency (plant)* – Level of benefit the plant receives from the mutualism.

*Multiple mutualists* (*plant*) – Plant host engages in multiple mutualisms.

*Dispersal limitation (mutualist)* – Ability of the mutualist partner to disperse (i.e. spread).

*Specificity (mutualist)* – Level of mutualist selectivity in the mutualism.

*Dependency (mutualist)* – Level of benefit that the mutualist receives from the plant.

Mutualisms can provide several vital services to their plant hosts, including defense, dispersal (or transportation), and nutritional benefits (Bronstein, 2015). Important examples can be found across the globe, from land to sea, from tropical forests to arctic tundra, and from intact to intensively managed systems. One textbook example of mutualism is the ant–acacia defense mutualism, where acacia trees form enlarged stipular thorns to house ants that defend them from herbivores (Janzen, 1967). Many plants also depend on biotic pollination for reproduction from animals as diverse as birds, bats, and insects. Animals also serve as important seed dispersal mutualisms (Janzen, 1985; Bronstein, 2015). Major nutritional plant mutualisms can be found belowground, involving microorganisms from two kingdoms: fungi and bacteria. The two most common nutritional mutualisms are with mycorrhizal fungi and nitrogen (N)‐fixing bacteria, with an estimated 90% of vascular plant species (Brundrett & Tedersoo, 2018) and most legumes (Franche *et al*., 2009) relying on these mutualisms, respectively. Beyond simple pairwise mutualisms, plants often leverage multiple mutualisms simultaneously (Primieri *et al*., 2022; Yamawo & Ohno, 2024), within and across guilds. This allows plant hosts to build a portfolio of benefits that can amplify services at once or over time and across environmental variation. Despite their established role in plant dispersal, reproduction, protection, survival, and growth, these mutualistic interactions remain largely overlooked across plant biogeographical research (Kreft & Jetz, 2007; Wiens, 2011; Cai *et al*., 2023). Specifically, little work has tested the relative importance of beneficial mutualistic interactions compared to historically studied abiotic and antagonistic effects.

Recent global synthesis work highlights the prominent influence mutualisms have on plant distributions (Simonsen *et al*., 2017; Delavaux *et al*., 2019, 2022, 2024; Taylor *et al*., 2019; König *et al*., 2021; Aizen & Torres, 2024). As plants rely on these partners, the successful establishment of their mutualistic partners may determine their success or failure. Several recent global analyses have shown that diverse mutualisms, from plant–mycorrhizal to biotic pollination, influence plant establishment (Delavaux *et al*., 2019, 2021, 2022, 2024; Taylor *et al*., 2019; König *et al*., 2021). The limited availability of these mutualists generates a filter (the mutualist filter) that limits plant host establishment. Support for this mutualist filter has also been found in regional and local analyses as well as experiments across the world (Koziol & Bever, 2017; Duchicela *et al*., 2020; Moyano *et al*., 2021). This initial mutualist filter has subsequent consequences for evolution and ecology, as well as implications for conservation, restoration, and invasions. Despite this emerging work showing the pervasive impact of the mutualist filter, mutualisms are still not systematically incorporated into our understanding of plant biogeography.

Given the fundamental role of mutualisms in influencing plant biogeography, a synthetic framework establishing the mutualism characteristics that mediate the strength of this filter is needed. Establishing such a framework will allow for the generation and testing of related hypotheses, the results of which should feedback to refine this framework and our understanding of the mutualist filter. In this review, I will (1) present evidence for the operation of the mutualist filter in island floras, arguing for its inclusion into plant biogeography (Section II); (2) discuss implications of this filter for ecology, evolution, and invasion from local to global scales (Sections III and IV); and (3) develop a framework for assessing the drivers, strength, and importance of the mutualist filter moving forward (Section V).

## II. Mutualist filters in island floras

**Fig. 1:**
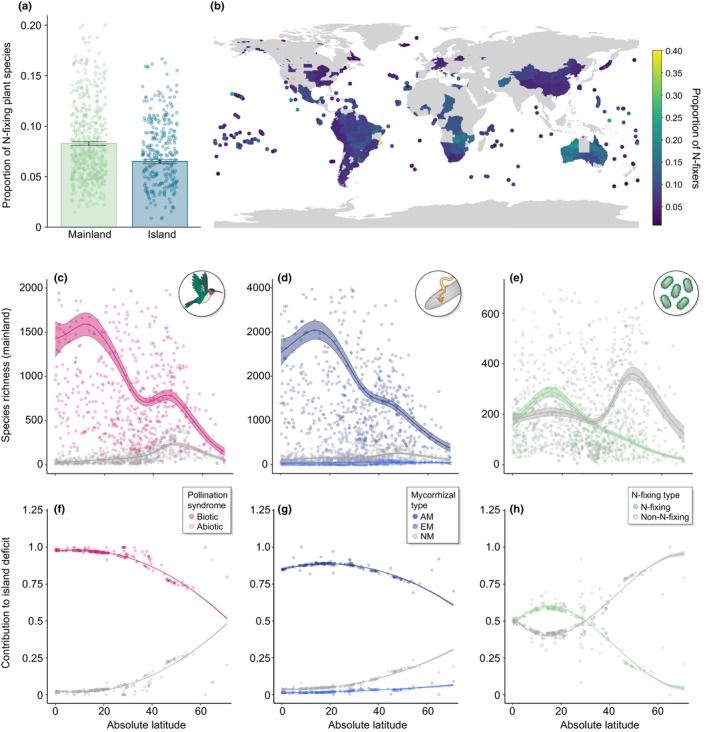
Mutualists influence plant biogeography. (a) Evidence for the mutualist filter within nitrogen (N)‐fixing plant species emerges as the proportion of N‐fixing plant species is lower on islands relative to mainlands; (b) data underlying (a) showing the proportion of N‐fixing plant species across included locations (Delavaux *et al*., 2022). (c–e) Across mainland systems, three major mutualisms – those between plant and biotic pollinators, arbuscular mycorrhizal (AM) fungi, and N‐fixing bacteria – are overrepresented at low latitudes. This mainland pattern results in a stronger mutualist filter (as measured by the proportion of species that fail to arrive on an island that are mutualist vs nonmutualist associated – *contribution to island deficit*) on low latitude islands (f–h, Delavaux *et al*., 2024). Error bars in (a) and shaded areas in (c–h) represent SEM.

The mutualism filter is consistent (Box 1), with numerous studies demonstrating that the absence or limited availability of these mutualistic partners can hinder plant establishment. This is evident from work on mainland range expansions being limited by mutualist dispersal (Fowler *et al*., 2023) and more recently by leveraging island systems (Delavaux *et al*., 2019, 2021, 2022, 2024; König *et al*., 2021; Schrader *et al*., 2021). Island systems have long inspired ecologists studying the drivers of plant biogeography (MacArthur & Wilson, 1967; Whittaker *et al*., 2017). These independent, replicated islands vary in important abiotic biogeographical variables and represent prime candidates for a mutualism filter due to their isolation. Work has shown that plant mutualists – including mycorrhizal fungi, N‐fixing bacteria, and plant pollinators – shape island plant biogeography (Fig. 1). Island plant establishment is limited by mycorrhizal fungi, a ubiquitous plant symbiont, and in particular, dispersal‐limited arbuscular mycorrhizal (AM) fungi (Delavaux *et al*., 2021). Moreover, this AM mutualist filter strengthens with distance to the mainland, consistent with the limited dispersal of AM fungi restricting plant host establishment (Delavaux *et al*., 2021). An analogous N‐fixing filter also acts on island plant establishment, with N‐fixing plant species underrepresented on islands world‐wide (Delavaux *et al*., 2022; Fig. 1a,b). Several independent assessments have shown that there is a similar biotic pollination mutualist filter, resulting in an island flora that is less biotically pollinated relative to the mainland (Kaiser‐Bunbury *et al*., 2010; König *et al*., 2021; Delavaux *et al*., 2024) and more self‐pollinated (Grossenbacher *et al*., 2017; Razanajatovo *et al*., 2019) (i.e. Baker's Law). Evidence for a filter in animal‐dispersed species is not as clear; however, work shows unspecialized dispersal syndromes are overrepresented on islands (König *et al*., 2021). Together, this work shows that the limitation of plant hosts by their mutualisms is a consistent feature shaping plant distribution patterns.

Despite the longtime focus of island biogeography on abiotic drivers of species richness (MacArthur & Wilson, 1967; Whittaker *et al*., 2023), recent research underscores the equally critical role of the mutualist filter. Not only is the mutualist filter general, but it is at least as important as classically studied abiotic physical island variables (Delavaux *et al*., 2024). Recent work showed that the plant mutualist filter, across mycorrhizal fungi, N‐fixing bacteria, and biotically pollinated plants, explained more variation in species richness on islands relative to traditional physical drivers of island biogeography (Delavaux *et al*., 2024). These results provide evidence that mutualisms generate a weakening of the latitudinal diversity gradient in species richness among oceanic islands, contribute to the maintenance of high tropical plant diversity, and mediate the biogeographic patterns of plant diversity on Earth. Therefore, it is time for island biogeography, and plant biogeography more generally, to recognize and integrate the role of mutualism.

## III. Implications for ecology and evolution

The legacy of this mutualist filter persists to impact island ecology and evolution. The operation of the mutualist filter results in island floras that are underrepresented in mutualist‐associated species. Remarkably, this pattern endures despite tens to hundreds of millions of years during which stochastic colonization events could have obscured it. Consequently, this mutualist filter contributes to island floras that are distinct from mainland sites, and in particular, that are less dependent on biotic interactions (Kaiser‐Bunbury *et al*., 2010; Grossenbacher *et al*., 2017; Delavaux *et al*., 2019, 2021, 2022, 2024; Razanajatovo *et al*., 2019; Taylor *et al*., 2019; König *et al*., 2021). Emerging evidence suggests that this initial distinction may have important implications for species diversification on islands. In particular, work focusing on mycorrhizal mutualism shows that diversification is highest for plants that experienced the strongest filter – specifically, those forming AM associations – and on more isolated islands where the filter is most pronounced. This was evaluated by examining the proportion of endemism on islands, species representing those that have likely evolved *in situ* on islands. These results show a greater proportion of endemic AM species at far islands (Delavaux *et al*., 2021). Similarly, research in pollinator and seed dispersal systems indicates that plants engaging in mutualisms show higher diversification rates (Xu *et al*., 2011; Weber & Agrawal, 2014; Larson‐Johnson, 2016). Certainly, more work is needed to test the role of mutualist association on species diversification patterns. Of particular importance will be testing for the competing influence of abiotic and biotic drivers on diversification.

## IV. Consequences for invasion risk

Although islands are ideal systems in which to test for the mutualist filter, mainland systems also illuminate evidence for a mutualist filter, with consequences for invasion risk. However, because the majority of native mainland systems have relatively high proportions of mutualist‐associated plants (Delavaux *et al*., 2024; Fig. 1c–e), the filter may emerge as a result of disturbance that disrupts mutualistic interactions (Moles *et al*., 2022; Aizen & Torres, 2024). For example, creation of a mutualist filter via disturbance can be seen in heavily degraded prairie systems, where degraded AM fungal communities (House & Bever, 2018) restrict restoration of highly mycorrhizally‐dependent late successional plant species (Koziol & Bever, 2017), even at a local scale. Disrupted seed dispersal due to disturbance can also limit plant distributions. For example, in Argentina, the distribution of mistletoe was affected by habitat fragmentation, disrupting seed dispersal by an endemic marsupial (Rodríguez‐Cabal *et al*., 2007). At larger scales, the invasion of pine trees into South America and New Zealand was successful only with the co‐invasion of their ectomycorrhizal (EM) fungal partners (Bunn *et al*., 2013; Yang *et al*., 2015; Reinhart *et al*., 2017; Moyano *et al*., 2020). Certain plant–pollinator systems suggest that plant invasion can be limited by the failure of both plant and pollinator to arrive. These include the failure of both *Ficus* and *Orchidaceae* species to become invasive, presumably due to the absence of specialized pollinators in novel ranges (Richardson *et al*., 2000; Taylor *et al*., 2019). Further global‐scale evidence for the impacts of the filter on invasion has been documented for N‐fixing plant species. A global analysis of N‐fixing plant species on mainlands showed that naturalized plant species are less likely to rely on N‐fixing bacteria (Simonsen *et al*., 2017). Nonetheless, such an assessment has not been conducted for the plant–mycorrhizal association, plant–pollinator, or seed dispersal associations. Research in the area of mutualist mediation of invasions is increasing (Richardson *et al*., 2000; Pringle *et al*., 2009; Dickie *et al*., 2017; Aizen & Torres, 2024), but the field is far from predictive. Clarifying the interactions between mutualisms and disturbance in driving invasion risk will be a top priority for mitigating the devastating impacts of plant invasions.

**Fig. 2:**
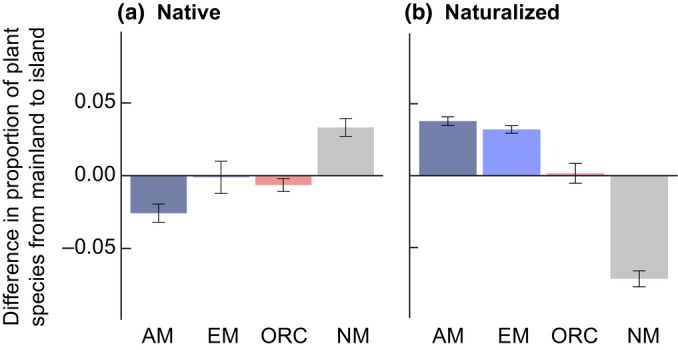
Invasions weaken the mutualist filter. (a) Native floras show an arbuscular mycorrhizal (AM) filter, with AM plant species underrepresented on islands relative to mainlands. However, (b) naturalized flora – plant species that are introduced and have established reproducing populations – no longer show this filter, with AM and ectomycorrhizal (EM) types overrepresented on islands (Delavaux *et al*., 2021). Other mycorrhizal plant types considered include orchid mycorrhizal (ORC) and nonmycorrhizal (NM) plant species. Error bars represent SEM.

In contrast to the mainland, on islands, mutualist‐associated species represent an elevated invasion risk (Fig. 2). World‐wide, mycorrhizal and N‐fixing plant species overcome initial mutualist filters, likely due to co‐introduction of plants with their symbionts (Delavaux *et al*., 2021; L. Muller *et al*., unpublished). This finding is extremely important in designing management and restoration targets for highly vulnerable and unique island systems (Fernández‐Palacios *et al*., 2021). This is especially striking with mycorrhizal plant species, with evidence from global analyses and local experiments. On mainlands, these are essential and highly abundant in native species, with invaders therefore more likely to succeed if they are nonmycorrhizal or less dependent (Pyšek *et al*., 2019). However, on islands, global‐scale modeling and experimental work show that nonnative or invasive plants tend to be more mycorrhizal (Delavaux *et al*., 2021) and more mycorrhizally dependent (Duchicela *et al*., 2020). A full understanding of how these initial reductions in mutualist plant species on islands (i.e. the mutualist filter) influence what types of plants pose an elevated invasion risk will enable more effective policies for the prevention of plant invasions and their consequences.

## V. Traits influencing the mutualist filter: a synthesis

**Fig. 3:**
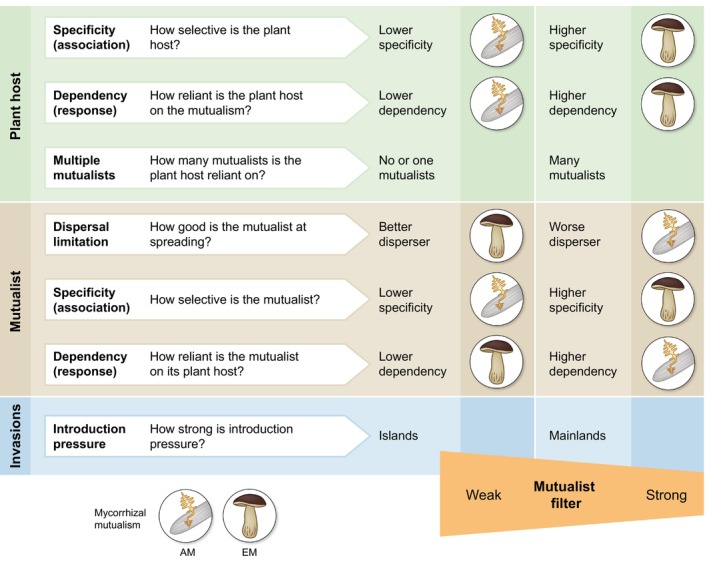
Major traits hypothesized to impact the mutualist filter. A framework synthesizing mutualism traits hypothesized to impact the strength of the mutualist filter. Plant host traits include specificity, dependency, and multiple mutualists; mutualist traits include dispersal limitation, specificity, and dependency. Finally, species introductions may mediate the filter, with differing effects on the mainland relative to islands. An example within the mycorrhizal symbiosis contrasting arbuscular mycorrhizal (AM) and ectomycorrhizal (EM) mutualisms is given in the dark gray boxes. Although EM mutualisms are relatively stronger in three out of five traits predicted to increase the mutualist filter, research shows that AM plant species experience a stronger filter, likely due to the overriding effect of dispersal limitation.

To study and predict the impact of mutualisms on plant biogeography, a unifying framework focusing on common traits that influence the strength of the mutualism filter across mutualisms is required. Here, I focus on such a framework to highlight important traits influencing the strength of the mutualism filter (Fig. 3; Box 1). These can be divided into mutualist vs plant (host) traits. Plant host traits include specificity, dependency, and association with multiple mutualists. Specificity describes the level of plant association specificity; dependency describes the level of benefit the plant receives from the mutualism (Chomicki *et al*., 2020); multiple mutualists describes the association of hosts with multiple mutualisms. Specificity and dependency are deliberately defined here to be used across mutualisms, and therefore may not correspond to field‐specific definitions (e.g. dependency here corresponds to mycorrhizal responsiveness, not dependency *sensu* (Janos, 2007)). Key mutualist traits include dispersal limitation, specificity, and dependence. Dispersal limitation describes the dispersal ability of the mutualist partner; specificity describes the level of mutualist association specificity; dependence describes the benefit the mutualist receives from the plant. The mutualist filter should strengthen where (1) the mutualist exhibits high dispersal limitation, (2) the plant and mutualist exhibit high specificity, and (3) the plant and mutualist show high dependency. As the mutualist is more dispersal limited, it is more likely to be absent when its host arrives; as the plant and mutualist exhibit high specificity, the chance of co‐arrival of the appropriate partners diminishes; as the plant and mutualist are more dependent on one another, timing of co‐arrival becomes more important because (A) as plant dependency increases, its ability to go without the mutualist declines and (B) as mutualist dependency increases, its ability to arrive and wait for its host declines. The importance of co‐arrival may decrease if the mutualist arrives and exhibits high dormancy or has the ability to be free‐living. The mutualist filter should further be amplified if the plant host has multiple mutualists with which it is associated (Taylor *et al*., 2019; Magnoli & Bever, 2023), as the co‐arrival of all partners becomes more limiting. Although this framework is developed with biotic pollinators, seed dispersers, mycorrhizal fungi, and N‐fixing bacteria as the mutualists in mind, it should be applied, tested, and adapted across other plant mutualisms.

These traits can be examined between major mutualist types, focusing on which mutualism should experience a stronger filter, or within a given mutualism. For example, certain pollinators may exhibit relatively high specificity (Johnson & Steiner, 2000), whereas most mycorrhizal associations (particularly AM) do not, predicting a stronger filter in these pollinator relationships relative to mycorrhizal relationships. Within mycorrhizal types, AM–plant associations should be relatively less specific (Crawford *et al*., 2019), but poorer dispersing than EM–plant associations (Peay *et al*., 2012), setting up competition hypotheses about which mycorrhizal type experiences a stronger filter. Based on previous work, dispersal seems to be the dominant driver within the mycorrhizal mutualism, as AM plant species show evidence of a stronger filter than EM plant species (Delavaux *et al*., 2021). At an even finer scale, AM fungal families may be expected to generate varying filter strengths. For example, AM fungal families show different investment in spore traits, including size (Chagnon *et al*., 2013; Chaudhary *et al*., 2025), resulting in differing dispersal ability, which could in turn influence expected filter strength. Smaller and more desiccation‐resistant spores, for example, should have a weaker filter than larger and more fragile spores. Future work should test these hypotheses and focus on which traits matter most to determine filter strength, with particular emphasis on traits that are consistent across mutualism types vs those that are mutualism‐specific. This work should span global, experimental, and theoretical approaches. Moreover, as mutualisms are known to be context‐dependent (Johnson *et al*., 1997), work expanding our understanding of the context dependence of the filter strength will be important. Finally, special attention should be given to assessing how plant and mutualist introductions alter the drivers and strength of the mutualist filter, and how this differs between mainland and island systems. Together, this will enable generalizations within the framework, while also allowing for a refined understanding of context‐ and mutualism‐specific drivers.

## VI. Conclusions

Mutualisms are pervasive and diverse, but show a common influence on plant distributions via the mutualist filter. That is, the absence of mutualists limits plant host establishment and spread. Here, I synthesize what is known about the mutualist filter and its consequences for ecology, evolution, and invasion across multiple mutualisms: mycorrhizal fungi, N‐fixing bacteria, pollinators, and seed dispersers. Finally, I present a unified framework to consolidate expectations of mutualist traits on the strength of the filter, for further study of the important role mutualisms play in plant biogeography.

## Competing interests

None declared.

## Disclaimer

The New Phytologist Foundation remains neutral with regard to jurisdictional claims in maps and in any institutional affiliations.
